# Editorial Comment: Anatomy of testicular artery: A proposal for a classification with MDCT angiography

**DOI:** 10.1590/S1677-5538.IBJU.2022.03.03

**Published:** 2022-04-14

**Authors:** Natasha T. Logsdon, Luciano A. Favorito

**Affiliations:** 1 Universidade do Estado do Rio de Janeiro Unidade de Pesquisa Urogenital Rio de Janeiro RJ Brasil Unidade de Pesquisa Urogenital - Universidade do Estado do Rio de Janeiro - Uerj, Rio de Janeiro, RJ, Brasil

Serife Balci ^1^, Selin Ardali Duzgun ^1^, Sevtap Arslan ^1^, Huseyin Balci ^2^, Musturay Karcaaltincaba ^1^, Ali Devrim Karaosmanoglu ^3^

Eur J Radiol. 2021 Sep;142:109885

## COMMENT

The knowledge of testicular arteries anatomy is important in pediatric urology and infertility surgeries. Previous studies about the testicular arteries in human fetuses shows a interesting pattern of anatomical variations (1, 2). During the Fowler-Stephens procedure in patients with high abdominal undescended testis is vey important this information is very important for the progress of the surgery (3). In the present paper the authors studied 400 patients during CT angiographies and (TAs) evaluated the number, origin, course, and caliber Testicular arteries showing some interesting anatomical variations. In more than 70% of the cases studied the testicular artery originates from the abdominal aorta, inferior to the level of renal artery. The authors shows a incidence of more than 25% of anatomical variations in anatomical pattern of testicular arteries. The figures in this paper are amazing and the authors concluded that normal anatomy and variations of testicular arteries may be effectively evaluated by CT angiography in a non-invasive manner.
